# Defining prerequisites for optimising outcomes of shoulder arthrodesis in post-traumatic brachial plexus palsy

**DOI:** 10.1007/s00590-026-04671-8

**Published:** 2026-02-11

**Authors:** G. Mithun Pai, Anil K Bhat, Ashwath M. Acharya, Kishore Vellingiri

**Affiliations:** Department of Hand Surgery, Kasturba Medical College, Manipal Academy of Higher Education, Manipal, India 576104

**Keywords:** Shoulder, Arthrodesis, Outcome, Brachial plexus, Function

## Abstract

**Purpose:**

Persistent pain, joint subluxation, instability, and limited shoulder mobility present significant reconstructive challenges in post-traumatic brachial plexus injury. This study aims to determine the essential prerequisites for attaining optimal functional results after shoulder arthrodesis in patients with post-traumatic brachial plexus palsy.

**Methods:**

Between 2017 and 2023, ten patients underwent shoulder arthrodesis, five with upper plexus palsy (mean age 35 years) and five with global palsy (mean age 31 years). The preoperative assessment included an evaluation of the integrity of the periscapular muscles. Arthrodesis was performed using a 3.5-mm locking reconstruction plate, positioning the shoulder in 30° forward flexion, 30° abduction, and 30° internal rotation. Patients were followed for a mean of 30 months, and outcomes were assessed in terms of radiological union, range of motion in terms of abduction, protraction, and retraction, brachiothoracic grasp, pain using the Visual Analog Scale and patient satisfaction using the Disabilities of the Arm, Shoulder, and Hand.

**Results:**

All patients achieved radiological union within an average of 8.5 months, with significant pain relief (mean VAS score: 1.1). Patients with global palsy demonstrated reduced mean forward flexion (38°) compared to those with upper palsy (68°). However, differences in abduction and external rotation were minimal. Functional gains were consistent across all patients, including improved brachiothoracic grasp, hand-to-mouth function, and stability.

**Conclusion:**

Shoulder arthrodesis remains a valuable salvage option for patients with multiple nerve avulsions or limited donor nerve availability. Optimal results with shoulder arthrodesis were observed in patients with intact or reanimated periscapular muscles. The procedure provides reliable pain relief and functional stability in post-traumatic brachial plexus palsy.

## Introduction

Restoring shoulder function remains a paramount objective in managing post-traumatic brachial plexus injury. Various surgical procedures, such as nerve reconstruction, tendon transfers, and shoulder arthrodesis, have been documented to improve stability and functionality of the shoulder. Among these, shoulder arthrodesis serves as a crucial, time-tested procedure in situations of persistent and profound paralysis, where alternative reconstruction offers limited advantages. It optimizes the resting position of the paralyzed shoulder, thereby improving spatial awareness and supporting compensatory movements. [1, 2] Glenohumeral arthrodesis results in pain alleviation and enhances scapulothoracic coordination [1]. A stable shoulder joint is crucial for optimal distal limb function; however, the effectiveness of arthrodesis in stabilizing a flail shoulder continues to be a subject of debate [3].

Nerve reconstruction procedures are reported to yield less predictable results for shoulder reanimation compared to the elbow, due to the joint’s intricate biomechanics. [4] The transfer of the trapezius alone could result in an awkward proximal displacement of the humeral head [5]. This resulted in mechanical impingement with a reduced trapezius lever arm, leading to suboptimal shoulder abduction and flexion [5, 6]. Although considerable gains in flexion and abduction were observed, the transfer often did not seem to resolve the external rotation insufficiency [6]. Enhancing shoulder external rotation should be considered with equal importance, as it enables patients to position their hand in front of their body, which often necessitates additional tendon transfers [3].

Despite advancements in tendon transfer procedures, residual impairments frequently persist, underscoring the importance of shoulder arthrodesis in achieving functional outcomes related to stability, pain relief, and facilitating clinically significant functions, such as the brachiothoracic grasp, particularly in cases of global palsy [7]. Although most literature on shoulder arthrodesis in brachial plexus palsy primarily emphasizes postoperative functional outcomes [8, 9], only a limited number of studies have explored the critical preoperative factors required to attain optimal results [10]. The primary objective of this study was to identify preoperative clinical factors that contribute to favourable functional outcomes after shoulder arthrodesis, with a particular focus on parascapular muscle function and optimal arthrodesis positioning. We hypothesized that the preservation of parascapular muscle function is associated with improved postoperative functional outcomes and that patients with upper plexus palsy demonstrate improved functional results following shoulder arthrodesis compared to those with global plexus palsy.

## Methodology

**Fig. 1:**
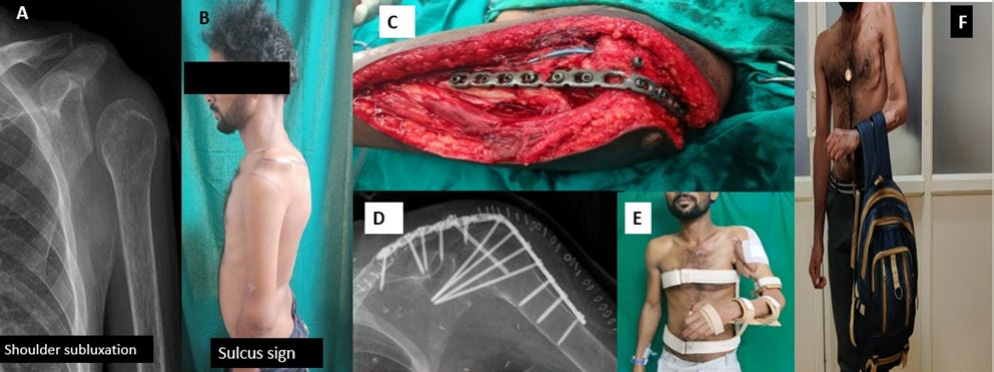
A patient with global brachial plexus palsy and an unstable shoulder. **A** Preoperative radiograph showing inferior subluxation of the humeral head. **B** Clinical photograph demonstrating a positive sulcus sign. **C** Intraoperative exposure of the shoulder with implant placement. **D** Postoperative radiograph showing screw configuration following arthrodesis. **E** Splint immobilization in the desired functional position. **F** One-year follow-up demonstrating regained shoulder stability

Based on the extent of the injury, patients were categorized into two groups: upper plexus palsy involving the C5–C6 or C5–C7 roots, and global palsy involving the C5–T1 roots. A total of 10 male patients with a mean age of 33.6 years (range, 23–65 years) underwent shoulder arthrodesis for post-traumatic brachial plexus palsy between January 2017 and December 2023 at our tertiary care referral centre. Among them, five had upper plexus palsy, and five had global palsy. Before arthrodesis, all patients had undergone various reconstructive procedures, including nerve transfers (e.g., intercostal to biceps, spinal accessory to suprascapular, Oberlin transfer), muscle transfers (e.g., gracilis for elbow flexion, latissimus dorsi to infraspinatus), or interfascicular nerve grafting. The primary indication for shoulder arthrodesis was a double-level suprascapular nerve injury in conjunction with axillary nerve involvement, in patients where no further nerve transfer or primary reconstruction was feasible, typically beyond 12 months post-injury, and who presented with a painful inferior glenohumeral subluxation (Fig. 1A, B). Institutional ethical clearance was obtained, and informed consent was obtained from all participants prior to the functional assessment.

### Surgical technique

The patient was placed in the beach-chair position under general anesthesia, and a standard deltopectoral approach was used to expose the glenohumeral joint (Fig. 1C). The articular cartilage of the glenoid, humeral head, and undersurface of the acromion was completely denuded to create raw, bleeding surfaces for bone-to-bone fusion—preoperative planning aimed for stable fixation with compression across both the glenohumeral and acromiohumeral joints. A 3.5 mm locking reconstruction plate was contoured and fixed laterally from the humeral shaft to the acromion and scapular spine, with at least four screws across the arthrodesis site. Additional fixation was provided by a 6.5 mm fully threaded cancellous screw passed transarticularly into the glenohumeral joint and, where feasible, a long-cannulated screw from the lateral acromion into the proximal humerus to assist acromiohumeral fusion (Fig. 1C, D). Corticocancellous bone graft harvested from the greater tuberosity, acromion, and/or coracoid was packed into the fusion bed to augment union. The shoulder was positioned in the functional 30° abduction, 30° flexion, and 30° internal rotation to optimize activities such as hand-to-mouth and midline reach before final tightening of the construct. Postoperatively, the limb was immobilized in a custom-moulded 30°–30°−30° splint for six weeks, followed by an arm sling and a structured rehabilitation program focusing on scapulothoracic mobility and periscapular muscle strengthening (Fig. 1E).

### Outcome assessment

Postoperative active shoulder abduction, forward flexion (protraction), and external rotation(retraction) were assessed clinically. Abduction angle and forward flexion (protraction) were measured in the frontal plane using the long axis of the humerus and the cervicothoracic spine forming the two limbs of the goniometer. External rotation was measured in the transverse plane with the elbow flexed to 90°, referencing the long axis of the forearm to a transverse limb of the goniometer that was parallel to the upper trunk [1]. We also included brachiothoracic grasp, which is assessed by observing the patient’s ability to stabilize objects between the upper arm and thoracic wall using scapulothoracic and elbow motion [10]. In addition, we assessed the ability to bring the hand to the mouth and rest on the belly, pain assessment using the Visual Analog Scale (VAS)[11], and patient-reported outcome measures using the DASH (Disabilities of the Arm, Shoulder, and Hand) score[12]. The motor status of the parascapular muscles were assessed clinically in each patient using standardized manual muscle testing to determine its impact on the outcome [13]. All measurement were assessed by two fellowship-trained upper limb surgeons with more than five years of experience.

### Statistical analysis

The mean and range for each variable were reported to summarize central tendencies and dispersion. To evaluate the differences in outcomes between patients with upper and pan-brachial plexus palsy, a combination of descriptive and inferential statistical methods was applied. Continuous variables, such as shoulder abduction, protraction, retraction, time to union, DASH scores (Disabilities of the Arm, Shoulder, and Hand), and VAS scores (Visual Analogue Scale for pain), were compared between the two groups using independent t-tests.

## Results

Patients (1–5) with upper brachial plexus palsy were predominantly young adults (age range, 23–65 years), with the right dominant upper limb affected in three cases (Table 1).

**Table 1:** The demographics of patients with brachial plexus injuries who underwent shoulder arthrodesis

Patient	Age	Gender	Side	Injury to the 1st surgery (month)	Ist surgery to arthrodesis (month)	Primary surgery	Reason for shoulder fusion	Final follow-up (month)
*Upper brachial plexus palsy*								
1	28	Male	Right	3	4	Oberlin	Double-level lesion (Suprascapular + Axillary nerve involvement)	16
2	23	Male	Left	2	7	Musculocutaneous nerve neurolysis	Double-level lesion (Suprascapular + Thinned axillary nerve)	12
3	65	Male	Right	5 (elsewhere)	24	Intercostal nerve to Musculocutaneous (Elsewhere)	Time since injury > 1 year (not amenable to repair)	12
4	26	Male	Right	12	6	Latissimus dorsi to Biceps	Time since injury > 1 year (not amenable to repair)	30
5	40	Male	Left	72	6	Latissimus dorsi to Biceps + Steindler	Time since injury > 1 year (not amenable to repair)	28
*Global brachial plexus palsy*								
6	32	Male	Right	6	7	Intercostal→Musculocutaneous, Intercostal→Pectoral, Free Gracilis (SAN)	Non-availability of nerve donors	72
7	36	Male	Right	6	6	Intercostal→Musculocutaneous, Intercostal→Pectoral, Free Gracilis (SAN)	Non-availability of nerve donors	14
8	28	Male	Right	7	4	Functioning Free Gracilis Transfer (SAN)	Time since injury > 1 year (not amenable to nerve repair)	36
9	31	Male	Right	7	8	Contralateral C7→Lower trunk, Free Gracilis Transfer (SAN)	Non-availability of nerve donors	36
10	27	Male	Right	2	6	Intercostal→Musculocutaneous, Intercostal→Pectoral, Free Gracilis (SAN)	Non-availability of nerve donors	36

The median interval from injury to first surgical intervention was approximately 5 months (range, 2–72 months), while the mean duration between the first Surgery and shoulder fusion was notably longer, averaging 11.8 months (range, 6–24 months). This delay likely reflects the period allowed for recovery following nerve reconstructions or muscle transfers. Initial reconstructive procedures varied and included Oberlin transfer, musculocutaneous nerve neurolysis, intercostal-to-musculocutaneous nerve transfer, and muscle transfers such as latissimus dorsi to biceps and Steindler’s flexorplasty. Shoulder arthrodesis was ultimately indicated for two primary reasons: in two patients, double-level lesions involving both the suprascapular and axillary nerves precluded shoulder stabilization, and in the remaining three patients, delayed presentation rendered further nerve reconstruction or transfer unfeasible. The follow-up duration ranged from 12 to 30 months (mean, 19.6 months), providing mid-term outcome data after fusion.

Patients (6–10) with pan-brachial plexus injuries underwent shoulder fusion primarily due to the limited reconstructive options available (Table 1). All were young males (age range, 27–36 years; mean, 30.8 years), with the right side affected in every case. The average time from injury to initial Surgery was 5.6 months (range, 2–7 months), while the mean interval between the first surgery and shoulder arthrodesis was 6.2 months (range, 4–8 months), reflecting a staged reconstruction approach. The primary interventions involved various combinations of nerve transfers, most commonly intercostal-to-musculocutaneous or intercostal-to-pectoral nerve transfers, and functioning free gracilis muscle transfers reinnervated by the spinal accessory nerve. One patient also underwent a contralateral C7 to lower trunk transfer. The principal indication for shoulder fusion in four of the five patients was the absence of viable donor nerves, making them unsuitable for further reconstructive procedures. The final follow-up period ranged from 14 to 72 months (mean, 38.8 months), allowing assessment of long-term functional outcomes.

**Fig. 2:**
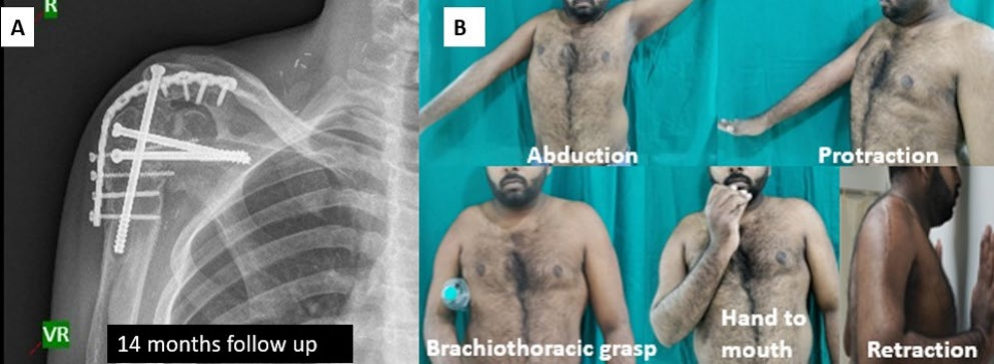
Patient with upper brachial plexus palsy showing **A** radiological outcome at 14 months demonstrating solid bony union and maintained alignment, and **B** functional outcome illustrating stable shoulder with satisfactory range of motion in the desired position

All patients with upper brachial plexus injuries who underwent shoulder arthrodesis retained a reasonable degree of scapulothoracic motion with a mean abduction of 52°, protraction of 68°, and a mean retraction of 46° (Table 2). All patients demonstrated recovery of functional upper limb independence with the ability to perform brachiothoracic grasp and hand-to-mouth activities. The mean time to union following shoulder fusion was 7.4 months, while the longest time to union was 11 months (Fig. 2).

**Table 2:** The functional outcomes of patients with upper brachial plexus injuries in comparison to those with pan-brachial plexus palsy who underwent shoulder fusion

Parameters	Upper brachial plexus palsy	Global brachial plexus palsy	Independent t-test
Abduction	52° (45°–60°)	44°(40°–45°)	*p* < 0.0001
Protraction	68° (60°–80°)	38° (30°–40°)	*p* < 0.0001
Retraction	46° (45°–50°)	41° (40°–45°)	*p* = 0.0003
Brachiothoracic grasp	Yes	Yes	
Hand to mouth/belly	Yes	Yes	
VAS Score	6.0 ± 5.5	16.0 ± 11.4	− *p* = 0.04
DASH Score	24.4 ± 4.5	35.8 ± 5.3	*P* = 0.02
Time to union	7.4 months (6–11 months)	7.2 months (6–9 months)	

We observed that patients with pan-brachial plexus palsy who underwent shoulder arthrodesis were able to demonstrate brachiothoracic grasp and hand-to-mouth activities, in addition to attaining considerable scapulothoracic motion despite profound neurological involvement. The scapulothoracic motion was demonstrated in terms of 44° degrees of shoulder abduction, 38° degrees of protraction, and 41° degrees of retraction, which contributed significantly to compensatory movement following fusion. Notably, these values were comparable to those observed in patients with upper brachial plexus injuries, implying that even in cases of pan-plexus involvement, a functional range of motion can be achieved when the periscapular muscles remain viable or are successfully reinnervated. The time to bone union after arthrodesis ranged from 6 to 9 months, consistent with expected healing timelines for shoulder fusion. These outcomes demonstrate meaningful functional gains following shoulder arthrodesis, reinforcing its role as an effective salvage procedure for complex brachial plexus injuries (Fig. 1F).

Statistical analysis comparing patients with upper brachial plexus palsy and pan brachial plexus palsy revealed significant differences across all outcome variables. Patients in the upper group exhibited significantly better abduction (*p* < 0.0001), protraction (*p* = 0.0003), and retraction (*p* = 0.0003) movements compared to the pan group (independent t-test). The time to union was comparable between the two groups. The DASH (Disabilities of the Arm, Shoulder, and Hand) score was significantly better in patients with upper plexus palsy (mean 24.4 ± 4.5) compared to those with global palsy (mean 36.8 ± 5.3), *p* = 0.004. Pain levels (VAS) improved in both groups, with no significant difference, indicating arthrodesis provided consistent pain relief regardless of palsy type (Table 2).

Patients with preserved or reinnervated pectoralis major, trapezius, serratus anterior, and rhomboid muscles, demonstrating a minimum of grade 3 muscle power, showed improved functional outcomes, as evidenced by lower DASH scores and minimal pain levels. In contrast, those with absent muscle power, particularly of the serratus anterior and pectoralis major, as noticed in Table 3.

**Table 3:** Parascapular muscle involvement across patients, along with their DASH scores (functional impairment) and VAS scores (pain)

Patient	Pectoralis	Trapezius	Serratus anterior	Rhomboids	DASH (100–0)	VAS (0–100)
*Upper brachial plexus palsy*						
1	No	Yes	Yes	Yes	26	0
2	Yes	Yes	Yes	Yes	24	0
3	Yes	Yes	Yes	Yes	31	10
4	Yes	Yes	Yes	Yes	20	10
5	Yes	Yes	Yes	Yes	21	10
*Global brachial plexus palsy*						
6	Yes	Yes	Yes	Yes	36	0
7	Yes	Yes	No	Yes	33	30
8	Yes	Yes	No	No	46	20
9	No	Yes	No	No	31	20
10	No	Yes	No	Yes	33	10

## Discussion

The primary conclusion of this study is that favourable functional outcomes following shoulder arthrodesis in cases of post-traumatic brachial plexus injury are predominantly associated with particular preoperative factors rather than with the surgical technique adopted alone. In particular, the preservation or successful reinnervation of critical parascapular muscles and achieving an optimal fusion position were strongly associated with improved postoperative function. These parameters appear essential in facilitating effective scapulothoracic compensation following the loss of glenohumeral mobility.

**Fig. 3:**
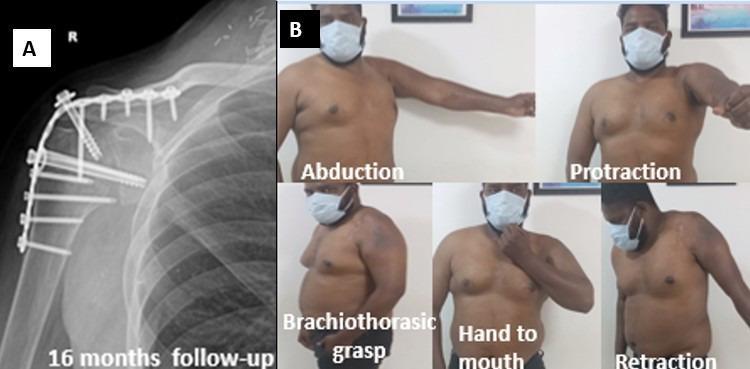
Patient with upper brachial plexus palsy showing **A** radiological outcome at 16 months demonstrating solid bony union and maintained alignment, and **B** functional outcome illustrating stable shoulder with satisfactory range of motion in the desired position

A comparative analysis of upper and global plexus palsy revealed that patients with upper plexus injuries typically exhibited improved range of motion and higher functional scores, likely due to the relatively preserved scapulothoracic mechanics. Notably, patients with global palsy exhibited considerable functional improvements, particularly in brachiothoracic grasp, alongside scapular protraction and retraction (Fig. 3), when the essential stabilizing muscles, such as the serratus anterior and pectoralis major, demonstrated a minimum of grade three muscle strength.

While the previous literature on shoulder arthrodesis has mainly concentrated on documenting postoperative function, limited studies have explored the factors that influence optimal outcomes. Garg et al. [14] and van der Lingen et al. [15] reported improved results in individuals with intact functionality of the spinal accessory nerve and scapulothoracic muscles, respectively. Nevertheless, these studies did not classify outcomes based on the pattern of plexus involvement with the exact extent of specificity as in the present series. Our findings suggest that classification based on neurological involvement provides enhanced prognostic insight in the context of arthrodesis.

The pectoralis major, trapezius, levator scapulae, serratus anterior, and rhomboids collaboratively facilitate scapulothoracic movement to compensate for diminished glenohumeral mobility. The trapezius and levator scapulae allowed arm elevation through scapular rotation, whereas the serratus anterior allowed forward reaching through protraction and upward rotation, despite the lack of deltoid function. The preservation or reinnervation of the pectoralis major was significantly associated with the recovery of brachiothoracic grip and forward reach, hence improving midline hand function. Patients with intact or reanimated parascapular musculature demonstrating a minimum of grade three muscle strength achieved better functional outcomes in our study, highlighting the need for comprehensive preoperative assessment and targeted nerve reconstruction (Table 3).

Functional results are also strongly influenced by fusion position. To enhance functional reach and minimize periscapular pain, Sousa et al. [16] recommended the fusion position of 35° of abduction, 30° of forward flexion, and less than 45° of internal rotation. Arthrodesis was performed in our series at approximately 30° of internal rotation, 30° of flexion, and 30° of abduction. This configuration has been associated with the most effective upper limb placement for everyday tasks while avoiding winging or severe scapular discomfort. Our findings are consistent with biomechanical evidence demonstrating balanced scapulothoracic loading. Furthermore, the simultaneous acromiohumeral and glenohumeral fusion was shown to boost fusion rates by Rehmann et al. [17], a concept that is integrated into our surgical technique.

A meta-analysis by Zhang et al. [18] comparing upper trapezius transfer and shoulder arthrodesis revealed similar outcomes for both treatments in terms of range of motion and complication rates. However, the analysis devoted little consideration to shoulder stability measures, brachiothoracic grasp, and scapular dynamics, all of which are crucial following arthrodesis.Our study aims to fill this gap by emphasizing the significance of scapular control and compensatory mechanisms, which have a direct impact on independence and long-term satisfaction beyond conventional outcome measures such as DASH or VAS scores.

Complications associated with shoulder arthrodesis include non-union, postoperative fracture, hardware irritation, and infection. Historically, non-union rates have reached as high as 20% [18], but improvements in fixation methods have significantly decreased this risk [17, 19]. In our series, the lack of significant complications can be attributed to stable fixation and optimal fusion positioning.

The study adhered to the STROBE guidelines [20] and aligns with the United Nations Sustainable Development Goals 3 and 4 [21]. However, the retrospective design and limited sample size restrict the generalizability of the findings, as well as other limitations. Given the retrospective design of the investigation, blinding was not practical. Objective measurement of parascapular muscle strength through electromyography or advanced imaging was not conducted; instead, assessments were based on clinical evaluation, which is subject to the examiner’s judgment and interpretation.. Further prospective investigations utilizing objective biomechanical assessment are recommended.

To conclude, shoulder arthrodesis continues to be a dependable salvage procedure for post-traumatic brachial plexus palsy when reconstructive options are limited. Favorable outcomes are closely associated with the preservation or reinnervation of essential parascapular muscles, notably the trapezius, serratus anterior, and pectoralis major, as well as correct fusion positioning. Even in cases of global plexus palsy, satisfactory functional recovery can be attained when scapulothoracic stability and compensatory movements are restored (Fig. 3).

## Data Availability

No datasets were generated or analysed during the current study.

## References

[1] Cho AB, Choi HJ, Ferreira CHV, YoshinobuKiyohara L, Bersani Silva G, Sorrenti L (2023) Shoulder arthrodesis for traumatic brachial plexus injuries: functional outcomes and complications. Hand (N Y) 18(1_suppl):6S-13S. 10.1177/1558944721998008 33880953 10.1177/1558944721998008 PMC9896286

[2] Kamineni S, Unger RZ, Desai R (2019) Shoulder arthrodesis in the management of glenohumeral pathologies. J Shoulder Elbow Arthroplasty. 10.1177/2471549219850655

[3] Elhassan B, Bishop AT, Hartzler RU, Shin AY, Spinner RJ (2012) Tendon transfer options for the shoulder in patients with brachial plexus injury. J Bone Joint Surg Am 94(15):1391–1398. 10.2106/JBJS.J.01913 22854992 10.2106/JBJS.J.01913

[4] Bertelli JA, Ghizoni MF (2007) Transfer of the accessory nerve to the suprascapular nerve in brachial plexus reconstruction. J Hand Surg Am 32(7):989–998. 10.1016/j.jhsa.2007.05.016 17826551 10.1016/j.jhsa.2007.05.016

[5] Elhassan B, Bishop A, Shin A, Spinner R (2010) Shoulder tendon transfer options for adult patients with brachial plexus injury. J Hand Surg Am 35(7):1211–1219. 10.1016/j.jhsa.2010.05.001 20610066 10.1016/j.jhsa.2010.05.001

[6] Bertelli JA (2010) Upper and lower trapezius muscle transfer to restore shoulder abduction and external rotation in longstanding upper-type brachial plexus palsies in adults. Microsurgery 31(4):263–267 21557304 10.1002/micr.20838

[7] Zhang D, Garg R, Earp BE, Blazar P, Dyer GSM (2021) Shoulder arthrodesis versus upper trapezius transfer for traumatic brachial plexus injury: a proportional meta-analysis. Adv Orthop 2021:4445498. 10.1155/2021/4445498 34691784 10.1155/2021/4445498 PMC8528632

[8] Cho AB, Choi HJ, Ferreira CHV, Yoshinobu Kiyohara L, Bersani Silva G, Sorrenti L (2023) Shoulder arthrodesis for traumatic brachial plexus injuries: functional outcomes and complications. Hand 18(1_suppl):6S-13S. 10.1177/1558944721998008 33880953 10.1177/1558944721998008 PMC9896286

[9] Thangarajah T, Lambert SM (2017) Glenohumeral arthrodesis for late reconstruction of flail shoulder in patients with traumatic supraclavicular brachial plexus palsy. Shoulder Elbow 9(4):266–271. 10.1177/1758573217693807 28932283 10.1177/1758573217693807 PMC5598819

[10] Chammas M, Goubier JN, Coulet B, Reckendorf GM, Picot MC, Allieu Y (2004) Glenohumeral arthrodesis in upper and total brachial plexus palsy: a comparison of functional results. J Bone Joint Surg 86-B:692–695 10.1302/0301-620x.86b5.13549 15274265

[11] Hayes MHS, Patterson DG (1921) Experimental development of the graphic rating method. Psychol Bull 18:98–99

[12] Hudak PL, Amadio PC, Bombardier C (1996) Development of an upper extremity outcome measure: the DASH (disabilities of the arm, shoulder, and hand) [corrected]. The Upper Extremity Collaborative Group (UECG). Am J Ind Med 29(6):602–608. 10.1002/(SICI)1097-0274(199606)29:6%3c602::AID-AJIM4%3e3.0.CO;2-L 8773720 10.1002/(SICI)1097-0274(199606)29:6<602::AID-AJIM4>3.0.CO;2-L

[13] Vijian K, Cheng YT, Idris Z, Izaini Ghani AR, Abdul Halim S, Abdullah JM (2023) Manual Muscle Testing of the Scapula and the Upper Limb through Bedside Examination. Malays J Med Sci 30(1):198–212. 10.21315/mjms2023.30.1.17 36875200 10.21315/mjms2023.30.1.17 PMC9984099

[14] Garg R, Malhotra R, Bhan S (2013) Glenohumeral arthrodesis in brachial plexus injury. J Shoulder Elbow Surg 22(6):792–799. 10.1016/j.jse22981352

[15] Van der Lingen MAJ, de Joode SGCJ, Schotanus MGM et al (2018) Satisfied patients after shoulder arthrodesis for brachial plexus lesions even after 20 years of follow-up. Eur J Orthop Surg Traumatol 28(6):1089–1094. 10.1007/s00590-018-2152-8 29453752 10.1007/s00590-018-2152-8 PMC6060881

[16] Sousa R, Pereira A, Massada M, Trigueiros M, Lemos R, Silva C (2011) Shoulder arthrodesis in adult brachial plexus injury: what is the optimal position? J Hand Surg Eur 36(7):541–547. 10.1177/1753193411405742 10.1177/1753193411405742 21490031

[17] Rühmann O, Schmolke S, Bohnsack M, Flamme C, Wirth CJ, Schmitt O (2005) Shoulder arthrodesis: indications, technique, and results. J Shoulder Elbow Surg 14(1):38–50. 10.1016/j.jse.2004.05.003 15723012 10.1016/j.jse.2004.05.008

[18] Cofield RH, Briggs BT (1979) Glenohumeral arthrodesis. Operative and long-term functional results. J Bone Joint Surg Am 61(5):668–677 457712

[19] Dimmen S, Madsen JE (2007) Long-term outcome of shoulder arthrodesis performed with plate fixation: 18 patients examined after 3-15 years. Acta Orthop 78(6):827–833 18236191 10.1080/17453670710014626

[20] Cuschieri S (2019) The STROBE guidelines. Saudi J Anaesth 13(Suppl 1):S31–S34. 10.4103/sja.SJA_543_18 30930717 10.4103/sja.SJA_543_18 PMC6398292

[21] Amorós Molina Á, Helldén D, Alfvén T et al (2023) Integrating the United Nations sustainable development goals into higher education globally: a scoping review. Glob Health Action 16(1):2190649. 10.1080/16549716.2023.2190649 36999571 10.1080/16549716.2023.2190649 PMC10071976

